# Diagnostic performance of serum 14-3-3η protein versus conventional serological markers in early rheumatoid arthritis

**DOI:** 10.3389/fimmu.2026.1746239

**Published:** 2026-02-12

**Authors:** Tiantian Liu, Wenbin Guo

**Affiliations:** 1 Department of Laboratory Medicine, Pingtan Comprehensive Experimental Area Hospital, Fuzhou, Fujian, China; 2 Department of Pathology, Pingtan Comprehensive Experimental Area Hospital, Fuzhou, Fujian, China

**Keywords:** 14-3-3η, biomarker, propensity score matching, ra, rheumatoid arthritis

## Abstract

**Objective:**

Conventional biomarkers for rheumatoid arthritis (RA) only have a ~70% sensitivity, making early diagnosis difficult. With its high concentration in synovial fluid, the 14-3-3η protein is a promising new biomarker for the early identification of RA in Chinese people.

**Methods:**

In this case-control study, 90 disease controls, 110 healthy controls, 72 established RA patients, and 56 early RA patients were enrolled. The 14-3-3η protein’s diagnostic performance was evaluated utilizing ROC curve analysis in comparison to anti-CCP antibodies, RF, CRP, IgM, and ESR. To control for confounding variables, multivariable logistic regression models that were adjusted for sex and age were used.

**Results:**

14-3-3η showed exceptional diagnostic performance, with AUC ≥0.85 for both early and developed RA. In early RA, anti-CCP demonstrated better specificity (96.4%) and a positive likelihood ratio (21.2%), while 14-3-3η had the highest sensitivity (88.1%) and the lowest negative likelihood ratio (0.14). There were no significant associations between 14-3-3η and traditional markers (P>0.05) according to Spearman correlation analysis.

**Conclusions:**

14-3-3η protein serves as an independent and highly sensitive biomarker for early RA diagnosis, particularly valuable for ruling out disease. These biomarkers, combined with the high specificity of anti-CCP, provide supplementary diagnostic benefits for complete early RA assessment.

## 1 Introduction

Rheumatoid arthritis (RA) is a chronic, inflammatory autoimmune disease (1) (2–4)characterized by joint invasion, predominantly affecting small joints in the distal extremities with symmetric distribution and recurrent episodes (5). In early stages, patients primarily present with redness, swelling, heat, pain, and stiffness in affected joints, while moderate to advanced stages manifest varying degrees of joint deformity accompanied by bone and skeletal muscle atrophy (6). During the chronic phase, synovial hypertrophy and hyperplasia occur within the joint cavity, forming pannus that destroys bone tissue and subsequently causes progressive joint dysfunction (7–9). More than 50% of RA patients develop varying degrees of joint bone destruction within two years of onset, ultimately leading to disability or even death (10). Research indicates that early diagnosis and timely treatment of RA can effectively prevent or significantly alleviate joint damage in 90% of RA patients (11).

Currently, the clinical diagnosis of rheumatoid arthritis primarily relies on the diagnostic criteria jointly established by the American College of Rheumatology (ACR) and the European League Against Rheumatism (EULAR) in 2010 (12), as shown in **Table 1**. This scoring system evaluates the number of affected joints, detects serological antibodies (RF, anti-CCP antibodies), acute phase reactants (CRP, ESR), and records the duration of synovitis, significantly improving the sensitivity and specificity of RA diagnosis (13–15). However, the sensitivity for early RA diagnosis remains unsatisfactory at approximately 70% (16). Therefore, it is necessary to explore the incorporation of new indicators to meet the diagnostic requirements for early RA.

**Table 1:** Index levels among groups.

	Early RA	Est RA	DC	HC	*P*
n	56	72	90	110	
Sex (Male/Female)	20/36	15/57	4/86	64/46	<0.001***
Age (median [IQR])	56.50 (48.00-64.00)	58.00 (51.75-67.25)	44.50 (33.75-54.00)	39.50 (28.00-52.00)	<0.001***
14-3-3η protein (median [IQR]) (ng/mL)	62.26 (47.75-99.22)	67.88 (53.12-114.16)	46.75 (33.95-69.16)	29.49 (22.77-37.76)	<0.001***
anti- CCP antibody (median [IQR])(U/mL)	23.85 (4.11-168.75)	112.00 (22.08-226.00)	0.50 (0.50-0.52)	0.34 (0.26-0.50)	<0.001***
RF (median [IQR]) (IU/mL)	180.00 (20.00-436.50)	222.50 (38.80-754.75)	20.00 (20.00-24.30)	20.00 (20.00-20.00)	<0.001***
CRP (median [IQR]) (mg/L)	13.40 (1.94-42.92)	15.49 (4.26-42.4)	3.88 (1.37-12.24)	2.49 (1.68-4.08)	<0.001***
ESR (median [IQR]) (mm/h)	32.00 (12.00-85.75)	32.50 (22.00-68.50)	21.50 (10.25-50.00)	8.00 (5.00-16.75)	<0.001***
IgA (median [IQR]) (g/L)	2.59 (2.26-3.33)	2.94 (2.04-4.12)	2.83 (2.27-3.32)	2.49 (1.80-3.07)	0.005**
IgG (median [IQR]) (g/L)	14.45 (11.50-17.20)	13.30 (11.00-17.35)	15.40 (13.33-19.57)	13.30 (12.00-14.83)	<0.001***
IgM (median [IQR]) (g/L)	1.60 (1.09-2.11)	1.52 (1.10-1.97)	1.03 (0.72-1.46)	1.14 (0.83-1.39)	<0.001***
C3 (median [IQR]) (g/L)	0.97 (0.88-1.07)	0.90 (0.79-1.07)	0.72 (0.54-0.89)	0.97 (0.84-1.05)	<0.001***
C4 (median [IQR]) (g/L)	0.21 (0.17-0.23)	0.20 (0.17-0.24)	0.13 (0.08-0.17)	0.21 (0.17-0.26)	<0.001***

**P < 0.01; ***P < 0.001.

The 14-3–3 proteins (products of the YWHA gene family) constitute a widely distributed and highly conserved family of acidic eukaryotic proteins with molecular weights of approximately 29–33 kd. First discovered by Moore et al. (17) in 1967 from neuronal proteins extracted from bovine brain tissue, this family comprises seven isoforms: α/β, γ, ϵ, σ, ξ, θ/τ, and η (18). The 14-3–3 proteins interact with over 200 intracellular ligands through “amphipathic grooves” (19, 20), participating in the regulation and control of multiple functions and disease mechanisms, including cell cycle regulation, transcriptional regulation, signal transduction, immune inflammation, and tumorigenesis (21, 22). Notably, the concentration of 14-3-3η protein in synovial fluid is five times higher than in serum (23), suggesting that 14-3-3η protein likely originates from synovial fluid.

Kilani et al. first reported the correlation between 14-3-3η protein and RA in 2007 (24). Their research team found that RA patients had significantly higher levels of 14-3-3η protein in both synovial fluid and serum compared to healthy individuals. This report also indicated a correlation between 14-3-3η protein and matrix metalloproteinases (MMPs). Subsequent studies revealed that as a pro-inflammatory mediator, 14-3-3η protein can participate in the regulation of various biological activities (25). Through MAPK/ERK, SAPK/JNK, and JAK-STAT signaling cascade pathways, it leads to upregulation of interleukin-1β, interleukin-6, tumor necrosis factor-α, joint-degrading factor MMP-9, and receptor activator of nuclear factor κB ligand, thereby promoting inflammatory joint damage. Additional reports indicate that extracellular 14-3-3η protein possesses ligand activity, activating macrophages and utilizing signal cascade effects to upregulate pro-inflammatory factors, effectively inducing inflammatory factor activation and leading to inflammation and even joint destruction (26).

Currently, most diagnostic studies on 14-3-3η protein for RA are based on European and American populations, with limited and conflicting research data available for the Chinese population. The study subjects are predominantly confirmed RA patients with disease duration exceeding one year, while data on early RA patients within one year of onset remain insufficient. This study aims to compare the diagnostic value of 14-3-3η protein with commonly used laboratory RA diagnostic indicators for early RA diagnosis and evaluate its diagnostic performance. This research can further provide clinical research value for the diagnosis and treatment of early RA patients in the Chinese population.

## 2 Materials and methods

### 2.1 Study population

A total of 128 rheumatoid arthritis (RA) patients were consecutively enrolled from Fujian Pingtan Comprehensive Experimental Zone Hospital between January and December 2023. Patients were classified into two groups based on the length of their disease: those with early RA (disease duration ≤1 year, n = 56) and those with established RA (disease duration >1 year, n = 72). All patients fulfilled the 2010 ACR/EULAR classification criteria for RA (12). Additionally, 90 patients with non-RA autoimmune diseases (60 with systemic lupus erythematosus and 30 with primary Sjögren’s syndrome) who met the respective diagnostic criteria were recruited as the disease control (DC) group. The healthy control (HC) group was a cohort of 110 healthy people who had no history of autoimmune disorders, infections, cancer, or hepatic dysfunction.

### 2.2 Laboratory assessments

Fasting venous blood samples were collected from each participant using vacuum tubes containing separation gel and 3.8% sodium citrate. Serum, isolated by centrifuging separation gel tubes at 3500 rpm for 15 minutes, was aliquoted and stored at -20 °C for subsequent analysis. Citrated whole blood was processed without pretreatment. Serum 14-3-3η levels were quantified using a commercial double-antibody sandwich ELISA kit (Hefei Laier Biotechnology Co., Ltd., China) according to the manufacturer’s protocol, with absorbance measured at 450 nm. Anti-CCP antibodies were assessed by chemiluminescent immunoassay (CLIA) on an iFlash 3000-C analyzer (Shenzhen YHLO Biotech Co., Ltd., China), with values >5 U/mL considered positive. Levels of RF, CRP, IgA, IgG, IgM, C3, and C4 were determined via immunonephelometry on an IMMAGE 800 system (Beckman Coulter, USA). The erythrocyte sedimentation rate (ESR) was measured in whole blood from RA patients, with results >20 mm/hour deemed elevated.

### 2.3 Statistical analysis

Data were analyzed using SPSS 23.0. Continuous variables with non-normal distribution are presented as median and interquartile range (IQR) and compared using the Kruskal-Wallis test. Categorical variables were compared by the chi-square test. Correlations between 14-3-3η and other parameters were examined using Spearman’s rank correlation. To mitigate confounding effects from baseline characteristics, propensity score matching was applied. Logistic regression models, adjusted for age and sex, were constructed. The diagnostic performance of biomarkers was evaluated by receiver operating characteristic (ROC) curve analysis, with the area under the curve (AUC), sensitivity (Se), specificity (Sp), Youden index (YDI), and likelihood ratios (+LR, -LR) calculated. A two-sided P-value < 0.05 was considered statistically significant.

## 4 Results

### 4.1 Baseline characteristics of the study population

After applying the inclusion and exclusion criteria, a total of 56 patients were enrolled in the early RA group, with ages ranging from 21 to 76 years and a median age of 56.50 years (**Table 1**). This group consisted of 36 females (64.29%) and 20 males (35.71%). The established RA (Est RA) group included 72 patients aged 18 to 84 years, with a median age of 58.00 years. Among them, 57 were female (79.17%) and 15 were male (20.83%). The disease control (DC) group comprised 90 patients with either systemic lupus erythematosus (SLE) or primary Sjögren’s syndrome (pSS), aged 18 to 75 years and with a median age of 44.50 years. The healthy control (HC) group consisted of 110 individuals aged 19 to 69 years, with a median age of 39.50 years, including 46 females (41.82%) and 64 males (58.18%). Statistically significant differences in age and gender distribution were observed among the groups (P < 0.001).

### 4.2 Assessment of the relationships between 14-3-3η and other biomarkers

To establish the novelty of 14-3-3η as a distinct biomarker, we evaluated its correlation with conventional serological markers. Spearman’s correlation analysis was performed to assess the relationship between 14-3-3η protein and other clinical parameters. As shown in **Table 2**, no significant correlations were observed between 14-3-3η and any of the other markers tested (P > 0.05), suggesting that 14-3-3η may serve as an independent diagnostic marker for rheumatoid arthritis.

**Table 2:** Correlation between 14-3-3η protein and other indicators.

	Early RA		Est RA	
	*P*	R^2^	*P*	R^2^
Age	0.53	0.08	0.14	0.17
Sex	0.79	0.03	0.28	0.12
Anti- CCP antibody	0.84	0.03	0.25	0.14
RF	0.23	0.16	0.15	0.17
CRP	0.52	0.09	0.46	0.09
ESR	0.20	0.18	0.84	0.02
IgA	0.97	-0.01	0.61	0.06
IgG	0.07	-0.24	0.28	0.13
IgM	0.87	-0.02	0.10	0.20
C3	0.80	0.04	0.89	0.02
C4	0.30	0.14	0.70	0.046

### 4.3 Propensity score matching to control for confounding variables

Due to significant differences in age and gender distribution among the groups (P < 0.001), propensity score matching was performed to minimize potential confounding effects on the experimental outcomes. Specifically, the early RA group and the established RA (Est RA) group were individually matched with the disease control (DC) group and the healthy control (HC) group, respectively. To identify biomarkers with consistent differential expression across multiple group comparisons, we defined a selection criterion: for the early rheumatoid arthritis (RA) group, we included only those biomarkers that exhibited statistically significant differences when compared separately with the disease control (DC) group and also when compared with the healthy control (HC) group. This selection process identified 14-3-3η protein, anti-CCP antibody, RF, CRP, and IgM. Similarly, for the established RA group, the same selection criterion was applied—significant differences in comparisons with both DC and HC groups—yielding a panel comprising 14-3-3η protein, anti-CCP antibody, RF, CRP, and ESR (**Table 3**).

**Table 3:** Analysis of differences between groups (after propensity score matching).

	Early RA (*P* value)		Est RA (*P* value)	
	DC	HC	DC	HC
Sex	0.555	0.77	0.077	1
Age	0.717	0.729	0.659	0.992
14-3-3η protein	0.042*	<0.001***	0.006**	<0.001***
Anti- CCP antibody	<0.001***	<0.001***	<0.001***	<0.001***
RF	0.007**	<0.001***	<0.001***	<0.001***
CRP	0.043*	0.024*	0.04*	<0.001***
ESR	0.641	<0.001***	0.04*	<0.001***
IgG	0.162	0.357	0.015*	0.204
IgA	0.011*	0.392	0.581	0.175
IgM	<0.001***	0.003**	<0.001***	0.081
C3	<0.001***	0.466	<0.001***	0.89
C4	<0.001***	0.245	<0.001***	0.112

*P < 0.05; **P < 0.01; ***P < 0.001.

### 4.4 Adjusting for age and sex in multivariable logistic regression models

Despite the application of propensity score matching to balance age and sex distributions, the resultant sample size was deemed insufficient for robust matched analysis. Consequently, to rigorously control for the potential confounding effects of these variables, we employed multivariable logistic regression models using the pre-matched, full cohort data. In these models, age and sex were included as covariates, together with the previously identified biomarker panels: for the early RA group (14-3-3η protein, anti-CCP antibody, RF, CRP, and IgM) and for the established RA group (14-3-3η protein, anti-CCP antibody, RF, CRP, and ESR). This approach allowed us to maximize statistical power while explicitly adjusting for residual confounding. The regression coefficients, odds ratios, and associated confidence intervals for each variable in the final models are comprehensively detailed in **Supplementary Tables S1**, **S2**.


Score=Intercept+∑(factors*coefficeint)+age*coefficient+sex*coefficient

### 4.5 Diagnostic performance of biomarkers in early RA

**Figure 1:**
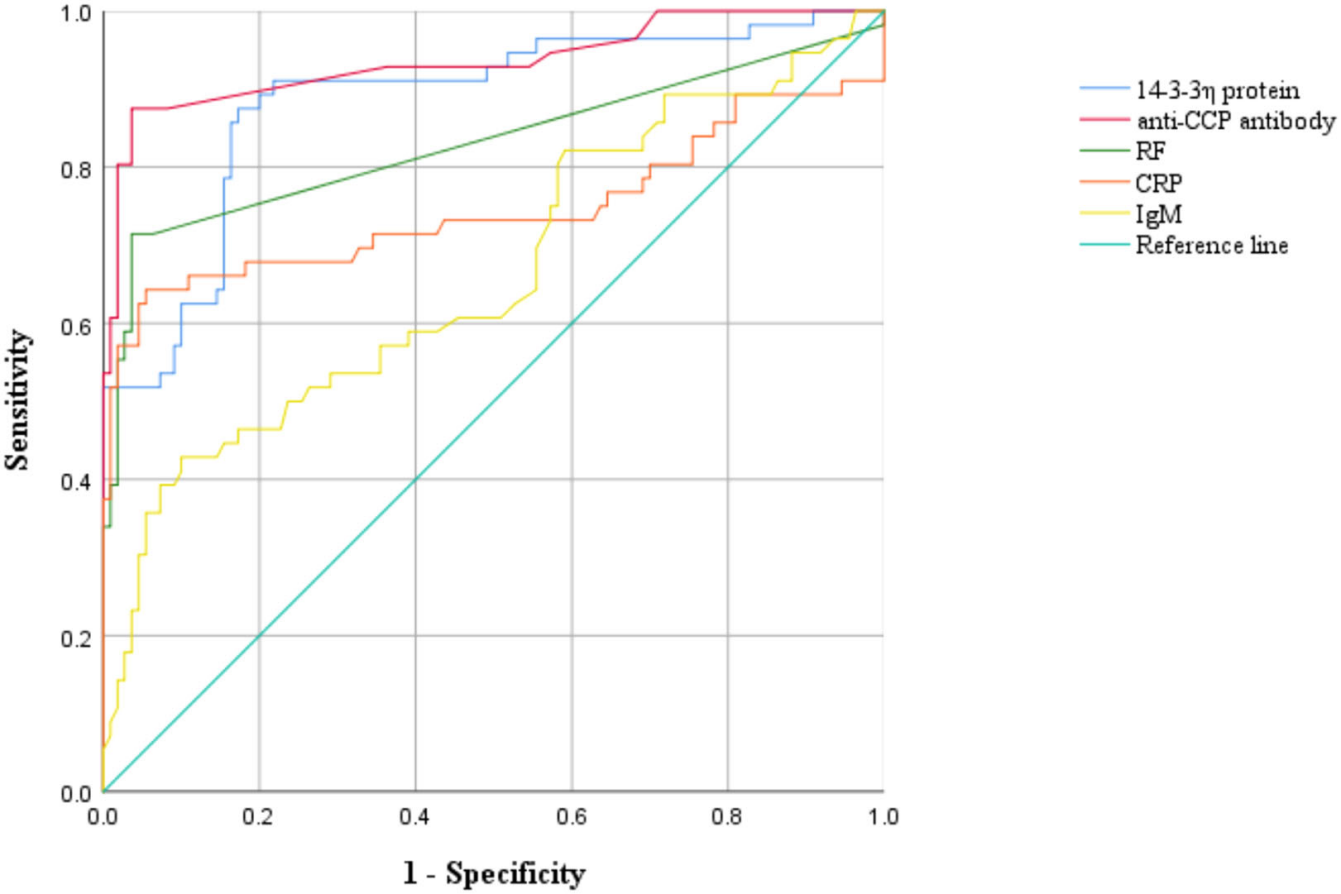
Receiver operating characteristic (ROC) curves of 14-3-3η protein, anti-cyclic citrullinated peptide antibody (anti-CCP), rheumatoid factor (RF), C-reactive protein (CRP), immunoglobulin M (IgM) and their combinations in early RA.

ROC curve analysis was utilized to evaluate and compare the diagnostic efficacy of 14-3-3η, anti-CCP, RF, CRP, and IgM in discriminating early RA patients from control subjects. The results demonstrated outstanding performance for both 14-3-3η and anti-CCP, each achieving an AUC ≥ 0.85. Specifically, 14-3-3η exhibited the highest sensitivity (88.1%) among all markers, along with a notably low negative likelihood ratio (0.14), highlighting its strength in ruling out early RA. Meanwhile, anti-CCP showed exceptional specificity (96.4%), the highest Youden index (0.81), and a markedly high positive likelihood ratio (21.25), supporting its utility in confirming diagnosis. RF and CRP displayed high specificities (91.4% and 94.6%, respectively), yet their sensitivities were considerably lower (71.4% and 64.3%), restricting their diagnostic value in early RA. IgM performed poorly across all metrics, with the lowest sensitivity (42.9%) and highest negative likelihood ratio (0.63) (**Table 4**; **Figure 1**), suggesting limited clinical relevance as a standalone marker.

**Table 4:** Clinical evaluation of 14-3-3η protein, anti-CCP, RF, CRP and IgM for the diagnosis of early RA.

	Cutoff value	AUC	Se%	Sp%	YDI	+LR	-LR
14-3-3η protein +Age+Sex	42.60	0.89	88.10	81.90	0.70	7.45	0.14
Anti- CCP antibody +Age+Sex	1.06	0.94	87.50	96.40	0.81	21.25	0.16
RF+Age+Sex	41.20	0.83	71.40	91.40	0.63	7.80	0.24
CRP+Age+Sex	7.88	0.75	64.30	94.60	0.59	12.80	0.38
IgM+Age+Sex	1.49	0.66	42.90	90.00	0.33	4.29	0.63

AUC, Area Under the Curve; Se, sensitive; Sp, specificity; YDI, Youden Index; LR, Likelihood Ratio.

### 4.6 Diagnostic performance of biomarkers in established RA

**Figure 2:**
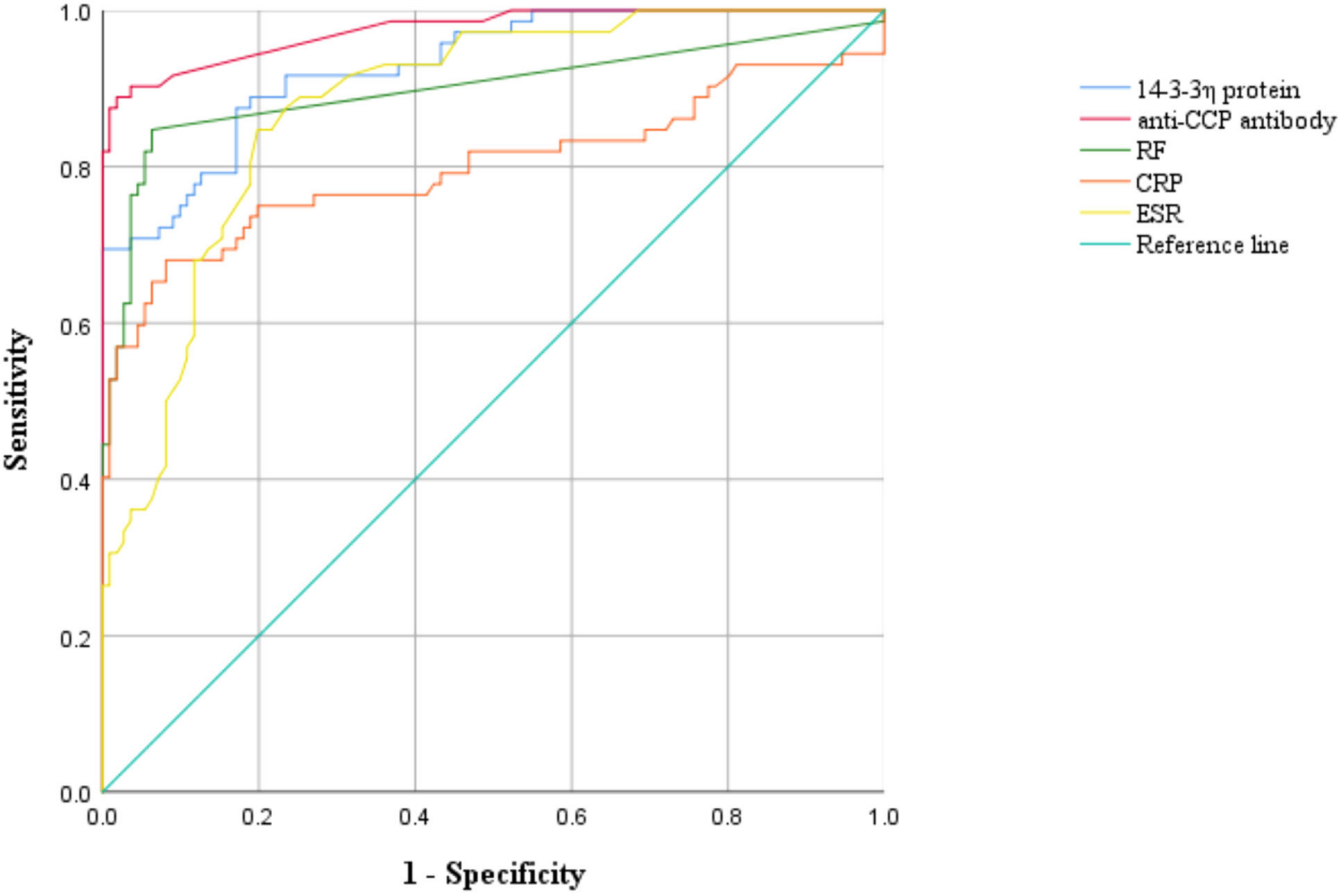
Receiver operating characteristic (ROC) curves of 14-3-3η protein, anti-cyclic citrullinated peptide antibody (anti-CCP), rheumatoid factor (RF), C-reactive protein (CRP), erythrocyte sedimentation rate (ESR) and their combinations in EST RA (established RA).

We further assessed the utility of 14-3-3η, anti-CCP, RF, CRP, and ESR in distinguishing established RA patients from controls. Both 14-3-3η and anti-CCP maintained high diagnostic accuracy, with AUCs exceeding 0.90 and sensitivities approximating 88–89%. Anti-CCP emerged as the superior marker in this group, achieving the highest specificity (98.2%), Youden index (0.87), and positive likelihood ratio (44.50), in addition to the lowest negative likelihood ratio (0.11). 14-3-3η displayed moderate specificity (83.4%) and a competitive negative likelihood ratio (0.14), reinforcing its role as a sensitive indicator. RF demonstrated a balanced profile with high sensitivity (84.7%) and specificity (93.6%), along with a robust positive likelihood ratio (14.17). In contrast, CRP exhibited the lowest sensitivity (68.1%) and a suboptimal negative likelihood ratio (0.35), whereas ESR showed the lowest specificity (80.0%) and a limited positive likelihood ratio (4.25) (**Table 5**; **Figure 2**), indicating that both markers offer comparatively lower discriminatory power in established RA.

**Table 5:** Clinical evaluation of 14-3-3η protein, anti-CCP, RF, CRP and ESR for the diagnosis of Est RA.

	Cutoff value	AUC	Se%	Sp%	YDI	+LR	-LR
14-3-3η protein +Age+Sex	41.71	0.93	88.60	83.40	0.72	5.18	0.14
Anti- CCP antibody +Age+Sex	3.83	0.98	88.90	98.20	0.87	44.5	0.11
RF+Age+Sex	20.65	0.90	84.70	93.60	0.79	14.17	0.16
CRP+Age+Sex	6.76	0.80	68.10	91.80	0.60	8.50	0.35
ESR+Age+Sex	19.50	0.88	84.70	80.00	0.65	4.25	0.19

## 5 Discussion

This study demonstrated that the 14-3-3η protein offers significant advantages in sensitivity (88.1%) and negative likelihood ratio (0.14) for early rheumatoid arthritis (RA) diagnosis compared to other biomarkers. Both 14-3-3η and anti-CCP antibody showed higher sensitivity and larger area under the curve (AUC) in both early and established RA groups than other indicators. RF and CRP exhibited high specificity but insufficient sensitivity, resulting in limited diagnostic value based on AUC. Although ESR showed some diagnostic relevance across all metrics, its performance was not prominent. IgM demonstrated low sensitivity and unsatisfactory AUC, rendering it unsuitable as an independent diagnostic marker for RA. After propensity score matching, IgA, IgG, C3, and C4 failed to show consistent statistical significance across both early and established RA groups compared to disease control (DC) and healthy control (HC) groups, and were therefore excluded from further diagnostic evaluation.

Pertsinidou et al. reported that the positivity rates of common diagnostic biomarkers varied significantly across RA patients of different ages and genders (27). Consistent with this, chi-square and ANOVA tests in our study revealed statistically significant differences in age and sex among the four participant groups (P < 0.001). To mitigate potential confounding effects from these variables, we applied propensity score matching to balance baseline characteristics and constructed logistic regression models to adjust for age and sex biases.

Maksymowych et al. reported that at a serum cut-off value of ≥0.19 ng/mL, 14-3-3η protein yielded a sensitivity of 77% and specificity of 93% for established RA (28). When applied to early RA, these values were 64% and 93%, respectively. In our study, both early and established RA groups showed higher sensitivity (88.1% and 88.6%, respectively) but lower specificity (81.9% and 83.4%) compared to those earlier findings. These discrepancies may be attributable to geographic, ethnic, and clinical differences, including treatment history among participants. Variations in assay manufacturers and antibody specifications may also contribute, especially since 14-3-3η has not yet been standardized as a commercial *in vitro* diagnostic marker.

Naides et al. observed that among 124 RA patients, 15 were anti-CCP negative, and 10 of those (67%) were positive for 14-3-3η, leading the authors to suggest that 14-3-3η may have superior clinical utility over anti-CCP (29). Our ROC analysis also indicated high sensitivity of 14-3-3η in both early and established RA, and the lowest negative likelihood ratio (0.14) in the early RA group, which is consistent with Naides’ findings. However, in our study, anti-CCP antibody demonstrated the highest specificity, Youden index, and positive likelihood ratio in both early RA (96.4%, 0.81, and 21.25, respectively) and established RA (98.2%, 0.87, and 44.50, respectively), along with the lowest negative likelihood ratio (0.11). Although 14-3-3η showed the highest sensitivity in early RA, its specificity and AUC were lower than those of anti-CCP. In established RA, anti-CCP and 14-3-3η had comparable sensitivity, but anti-CCP showed better specificity and higher AUC. Therefore, it remains uncertain whether 14-3-3η offers greater clinical utility than anti-CCP. Nonetheless, 14-3-3η demonstrates excellent sensitivity, especially in early RA.

Maksymowych et al. reported no correlation between 14-3-3η and CRP or ESR (P > 0.05) (30), a finding corroborated in our study for both early and established RA groups. The lack of correlation, combined with high sensitivity and AUC values of 14-3-3η in both RA groups, supports its potential as an independent diagnostic marker for RA.

As a retrospective study, our research is limited by non-randomized case selection, which may introduce selection bias. Future studies should incorporate synovial fluid 14-3-3η levels across different RA stages, along with imaging data such as X-ray of hands and wrists, swollen joint count (SJC28), tender joint count (TJC28), and other clinical indices to further evaluate the diagnostic utility of 14-3-3η throughout the disease course.

## 6 Conclusion

Serum 14-3-3η protein shows considerable promise as a novel diagnostic biomarker for RA. It complements existing biomarkers, improves diagnostic accuracy, and facilitates early RA detection, warranting broader clinical attention and application. Further multi-center studies with larger sample sizes are needed to validate its diagnostic utility, accounting for geographic, ethnic, environmental, and therapeutic variables, to scientifically establish its clinical value through large-scale data analysis.

## Data Availability

The raw data supporting the conclusions of this article will be made available by the authors, without undue reservation.
