# Supplementary material for: Diagnostic performance of serum 14-3-3η protein versus conventional serological markers in early rheumatoid arthritis
Source: Front Immunol. 2026 Feb 12;17:1746239. doi: 10.3389/fimmu.2026.1746239 (PMC12935600; doi:10.3389/fimmu.2026.1746239)
Supplement: Supplementary file 1 [file DataSheet1.docx]

**Supplementary table 1.** Coefficients of each index in the Early RA group

|  | coefficient | | | | |
| --- | --- | --- | --- | --- | --- |
|  | 14-3-3η protein +Age+Sex | anti- CCP antibody +Age+Sex | RF  +Age+Sex | CRP  +Age+Sex | IgM  +Age+Sex |
| Intercept | 12.49 | 14.46 | 12.41 | 6.79 | 5.91 |
| Age | 0.86 | 0.79 | 0.84 | 0.90 | 0.90 |
| Sex | 1.04 | 15.64 | 22.87 | 1.58 | 6.23 |
| 14-3-3η protein | 0.90 |  |  |  |  |
| anti- CCP antibody |  | 0.59 |  |  |  |
| RF |  |  | 0.90 |  |  |
| CRP |  |  |  | 0.91 |  |
| IgM |  |  |  |  | 0.37 |

**Supplementary table 2.** Coefficients of each index in the Est RA group

|  | coefficient | | | | |
| --- | --- | --- | --- | --- | --- |
|  | 14-3-3η protein +Age+Sex | anti- CCP antibody +Age+Sex | RF  +Age+Sex | CRP  +Age+Sex | ESR  +Age+Sex |
| cutoff value | 12.40 | 8.09 | 11.05 | 3.64 | 5.41 |
| Age | 2.44 | 0.89 | 0.86 | 0.94 | 0.92 |
| Sex | 14.88 | 156.02 | 12.68 | 20.09 | 4.31 |
| 14-3-3η protein | 0.87 |  |  |  |  |
| anti- CCP antibody |  | 0.63 |  |  |  |
| RF |  |  | 0.90 |  |  |
| CRP |  |  |  | 0.82 |  |
| ESR |  |  |  |  | 0.94 |
